# Larval superiority of *Culex pipiens* to *Aedes albopictus* in a replacement series experiment: prospects for coexistence in Germany

**DOI:** 10.1186/s13071-018-2665-3

**Published:** 2018-02-02

**Authors:** Ruth Müller, Timm Knautz, Simone Vollroth, Robert Berger, Aljoscha Kreß, Friederike Reuss, David A. Groneberg, Ulrich Kuch

**Affiliations:** 1 Goethe University Frankfurt am Main, Institute for Occupational Medicine, Social Medicine and Environmental Medicine, Theodor-Stern-Kai 9, 60590 Frankfurt am Main, Germany; 2 Goethe University Frankfurt am Main, Institute of Ecology, Evolution and Diversity, Max-von-Laue-Straße 13, 60438 Frankfurt am Main, Germany; 3 Senckenberg Biodiversity and Climate Research Centre, Senckenberganlage 25, 60325 Frankfurt am Main, Germany

**Keywords:** Microalgae, Interspecific competition, Niche differentiation, Resource competition, Water chemistry

## Abstract

**Background:**

The Asian tiger mosquito *Aedes albopictus* is an extremely invasive, globally distributed and medically important vector of various human and veterinary pathogens. In Germany, where this species was recently introduced, its establishment may become modulated by interspecific competition from autochthonous mosquito species, especially *Culex pipiens* (*s.l*.). While competitive superiority of *Ae. albopictus* to *Cx. pipiens* (*s.l*.) has been described elsewhere, it has not been assessed in the epidemiological conditions of Germany. The present study aimed to determine if such superiority exists under the physicochemical and microclimatic conditions typical for container habitats in Germany.

**Methods:**

In a replacement series experiment, the larval and pupal responses of *Ae. albopictus* and *Cx. pipiens* (*s.l*.) (mortality, development time, growth) to interspecific interaction (five larval ratios) at (sub-)optimal temperatures (15, 20 and 25 °C) and differing food supply (3 and 6 mg animal-based food larva^-1^) were investigated using a randomized split-plot design. In addition to physicochemical measurements of the test media, natural physicochemical conditions were determined for comparative analyses in mosquito breeding sites across the Rhine-Main metropolitan region of Germany.

**Results:**

Under the physicochemical and microclimatic conditions similar to the breeding sites of the Rhine-Main region, competitive superiority of *Cx. pipiens* (*s.l*.) to *Ae. albopictus* in terms of larval survival was more frequently observed than balanced coexistence. Food regime and multifactorial interactions, but not temperature alone, were controlling factors for interspecific competition. Larval food regime and the larval ratio of *Ae. albopictus* influenced the physicochemistry and algal growth at 15 °C, with increased *Ae. albopictus* mortality linked to a decreasing number of *Scenedesmus*, *Oocystis* and *Anabaena* algae.

**Conclusions:**

Under the present environmental conditions, the spread of *Ae. albopictus* from isolated foci in Germany may generally be slowed by biotic interactions with the ubiquitous *Cx. pipiens* (*s.l*.) (and potentially other container-breeding mosquito species) and by limnic microalgae in microhabitats with high resource levels. Detailed knowledge of the context dependency in temperate mosquito ecology, and interrelations of physicochemistry and phycology may help to achieve a better understanding of the upcoming *Ae. albopictus* colonization processes in central and northern Europe.

**Electronic supplementary material:**

The online version of this article (10.1186/s13071-018-2665-3) contains supplementary material, which is available to authorized users.

## Background

The extremely invasive mosquito *Aedes* (*Stegomyia*) *albopictus* Skuse 1894, a known vector of various human pathogens like dengue and chikungunya viruses, is gradually extending its distribution to colder climate regions [1]. At present, the northernmost breeding populations of this species in Europe occur in Freiburg [2], Heidelberg and Jena, Germany (Norbert Becker, personal communication, June 2016). A further spread of *Ae. albopictus* into the Rhine rift valley and parts of Bavaria and North Rhine-Westphalia, Germany, is very likely due to climatic habitat suitability and man-made features [3, 4]. Apart from the frequently suboptimal low temperatures in these regions, the establishment of *Ae. albopictus* may be modulated by interactions with resident container-breeding mosquitoes [5–7].

*Aedes albopictus* was shown to be a strong competitor for resident mosquito species such as *Aedes triseriatus* as well as exotic species like *Aedes aegypti* in the United States of America [7, 8]. However, environmental variation may change competitive balances [9] as recently demonstrated in Japan [10]: At Mount Konpira, Japan, the formerly predominant *Ae. albopictus* is currently becoming superseded by the formerly rare, but now superior competitor *Aedes flavopictus*, most probably due to changes in differential responses to environmental variability and altered species interactions [10]. As the competitive superiority of container-breeding mosquitoes strongly depends on context and is reversible, resident mosquitoes may have the potential to act as superior competitors in colder ecoregions due to their better adaptation to low temperatures than the invader *Ae. albopictus* which has a (sub-) tropical origin [11].

Negative interactions between different mosquito species frequently occur during their aquatic larval developmental stages and can be attributed to direct (chemical/physical interference) or indirect interactions (resource competition), generally known as interspecific competition [7, 9, 12]. Numerous laboratory investigations have corroborated that the pattern of the heterospecific larval response of *Ae. albopictus* depends not only on the competitor but also on the environmental context [9], food quantity or quality [5, 13–17] and food-temperature interactions [18, 19]. Larval resource competition has been considered as a probable reason for the decline of *Ae. aegypti* in concurrence with the arrival of *Ae. albopictus* in the United States [15, 20]. Furthermore, species-specific differences in resource utilization are thought to shape microhabitat-specific mosquito assemblages [15, 16, 21]. Water chemistry, as a direct function of available food resources and resource utilization by mosquitoes, has also been considered to control niche differentiation [22–24]. For instance, concentrations of nitrite and nitrate and the pH shaped the local distribution of the exotic *Aedes notocriptus* and the endemic *Culex pervigilans* in New Zealand [24]. Furthermore, certain microalgal species have been shown to exclude *Ae. albopictus* from microhabitats in Hawaii [22]; consequently, microalgae have been recommended as agents of biological mosquito control [25].

The common house mosquito *Culex pipiens* (*s.l*.) Linnaeus 1758 is a competent vector for West Nile and Usutu viruses and other viral and parasitic pathogens of human and veterinary importance [26]. This species, in particular, could be a strong competitor for *Ae. albopictus* in Germany because both species often share breeding containers [18]. In the laboratory, a superiority of *Ae. albopictus* over *Cx. pipiens* (*s.l*.) has been demonstrated [18, 27]. A significant niche overlap of *Ae. albopictus* and *Cx. pipiens* (*s.l*.) has been confirmed in Italy (11.3% of 1194 monitored containers) although habitat preferences differed slightly in terms of container volume (*Ae. albopictus*: < 5 l; *Cx. pipiens* (*s.l*.): > 5 l) and microclimate (*Ae. albopictus*: shaded sites; *Cx. pipiens* (*s.l*.): sunny sites) [18]. Both species overlapped greatly in seasonal population growth [18, 27, 28] whereas thermal preferences differ (*Ae. albopictus*: 29.7 °C [29], *Cx. pipiens* (*s.l*.): 20 to 25 °C [30]). The overlapping ecological niche and seasonal population activities in the presence of dissimilar autecological optima render a regionally disparate competitive superiority of *Ae. albopictus* over *Cx. pipiens* (*s.l*.) more than likely [5, 18, 27].

The ecology of container-breeding mosquitoes in temperate regions is still poorly understood. To achieve a better mechanistic understanding of the upcoming *Ae. albopictus* colonization processes in Germany, we tested whether the described competitive superiority of *Ae. albopictus* over *Cx. pipiens* (*s.l*.) also exists under the physicochemical and microclimatic conditions that are typical for container habitats in the Rhine rift valley of Germany. We hypothesized that (i) competitive superiority depends on the species-specific thermal tolerance spectrum, and therefore *Ae. albopictus* might be advantaged at higher and *Cx. pipiens* (*s.l*.) at lower temperatures, respectively. We further hypothesized that (ii) food regime, as a major determinant for interspecific interactions, interacts with temperature and that (iii) resource competition between *Ae. albopictus* and *Cx. pipiens* (*s.l*.) is directly connected to physicochemical and phycological parameters of their microhabitat.

## Methods

### Natural microhabitats of mosquito larvae

#### Physicochemistry

In a radius of 50 km around the city of Frankfurt am Main (Rhine-Main Metropolitan region, northern border of the Rhine rift valley, Germany), intermittent and permanent waterbodies of natural and anthropogenic origin (natural: temporary and permanent puddles, flooded patches/pools in forests and meadows, tree holes, root and rock cavities; anthropogenic: agricultural plastic covers, troughs on paddocks, water storage basins, fountain basins, planters, vases, barrels, car tires, buckets, watering cans) were monitored for the occurrence of mosquito larvae from 27th August to 27th October 2010 (Additional file 1: Figure S1).

#### Species identification

Species identification was based on sequence analysis of the ‘barcode region’ of the mitochondrial cytochrome *c* oxidase subunit 1 (*cox*1) gene. Following proteinase K digestion and phenol-chloroform extraction [31], the DNA of larvae was used for PCR amplification [TrueStartTM Hot Start-*Taq* DNA polymerase (Fermentas, St. Leon, Germany), 5 pmol LCO1490 primer, 5 pmol HCO 2198 primer, MWG Operon (Eurofins, Ebersberg, Germany)] [32]. After Sanger sequencing using the amplification primers, the ~ 700 bp *cox*1 mitochondrial DNA barcodes were submitted to the following databases, and compared to entries therein for species identification: Barcode of Life Data System (boldsystems.org), NCBI-BLAST (http://blast.ncbi.nlm.nih.gov/Blast.cgi) and European Nucleotide Archive (ebi.ac.uk/ena).

At a subset of sites positive for *Cx. pipiens* (*s.l*.), conductivity (± 2%, LWT-01 sensor, Voltcraft, Hirschau, Germany), temperature (± 0.8 °C, DO-100 sensor, Voltcraft, Hirschau, Germany), oxygen concentration (± 0.4 mg l^-1^, DO-100 sensor, Voltcraft, Hirschau, Germany) and pH (± 0.01, PHT-02 ATC sensor, Voltcraft, Hirschau, Germany) were measured *in situ*. Also, 1 L plastic cups were filled with 600 ml water and placed at a sunny and a shaded site in Frankfurt am Main. The water temperature in the transparent containers was recorded at 20-min intervals (64K Pendant® Data Logger UA-002-64, HOBO, OneTemp Pty Ltd., Marleston, Australia) from mid-May to the end of July 2011. During this period, a minimum water volume of 600 ml was maintained.

### Replacement series experiment

#### Experimental design

The interspecific larval interaction of *Ae. albopictus* and *Cx. pipiens molestus* under different food regimes and temperatures was investigated in a replacement series experiment using a randomized split-plot design. The replacement series experiment with 4 replications was designed to test the impact of the whole plot factors ‘species’ (2 levels) × ‘larval ratio’ (5 levels) × ‘temperature’ (3 levels) and the subplot factor ‘larval food regime’ (2 levels) on survival, development time and growth of the aquatic life stages of the 2 species.

#### Mosquito material

The mosquitoes used in the experiments were purchased from Biogents AG (Regensburg, Germany) and reared in-house. We used 2 well-adapted long-term laboratory strains (*Ae. albopictus* strain with origin Singapore, *Cx. pipiens* biotype *molestus* with origin Regensburg, Germany) to minimize unknown impacts of the field histories of the mosquito strains on their responses and better determine the fundamental nature of the context-dependency of interspecific interactions.

The eggs of *Ae. albopictus* were collected on filter paper, dried at 20 °C and 90% relative humidity, and exposed to a yeast solution stimulating larval hatching [33]. First-instars of *Cx. pipiens molestus* were released from egg rafts into 1:1 tap: deionized water (hereafter called larval medium). In total, 3600 larvae were investigated.

First-instar larvae of the two species, which had been synchronously released within 24–48 h, were allotted as pure or mixed cohorts of 30 larvae in 1 l test vessels (plastic cups) filled with 600 ml larval medium each.

#### Larval ratio

In total, 2 pure and 3 mixed larval groups were tested at 3 temperatures and under 2 food conditions. Following the recommendations of Oberg et al. [34], experimental units with only *Ae. albopictus* larvae (Ae: Cx^30:00^) and only *Cx. pipiens molestus* larvae (Ae: Cx^00:30^) and symmetrically and asymmetrically mixed treatments with a 20:10, 15:15 or 10:20 distribution of *Ae. albopictus*: *Cx. pipiens molestus* larvae (Ae: Cx^20:10^, Ae: Cx^15:15^, Ae: Cx^10:20^) were prepared. The larval density of 50 larvae l^-1^ reproduced natural larval densities: for instance, 1.2–89.5 larvae l^-1^ had been reported from various containers in Italy [18], 8.3–3080 larvae l^-1^ in tires in Florida [35], 1.4–80 larvae l^-1^ in buckets in North Carolina [36] and 0.6–62.4 larvae l^-1^ in tires and tree holes in Mississippi, USA [37].

#### Temperature

**Fig. 1:**
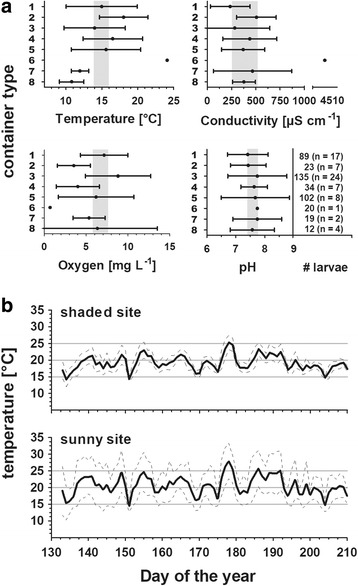
Physicochemistry in established and potential larval mosquito microhabitats. **a** Physicochemistry in natural habitats of *Cx. pipiens* in the periphery of Frankfurt am Main, Germany, during September and October 2010. Temperature (°C), pH, conductivity (μS cm^-1^) and dissolved oxygen concentration (mg l^-1^) (mean ± SD) and respective *Cx. pipiens* abundance [# larvae: number of larvae (*n* = number of microhabitats)] are shown. Grey bars denote overall 95% confidence intervals. *Key*: 1, car tires; 2, vases; 3, rain barrels; 4, small reservoirs (bucket, plastic tarpaulin, pot, steel girder); 5, large reservoirs (metal and plastic tub, excavator shovel, water bowl); 6, dung discharge; 7, puddles; 8, ponds. **b** Spline curve of daily water temperature [mean ± SD] in 1 litre plastic cups logged from mid-May to end of July 2011. The cups representing potential mosquito habitats had been set up in shaded *versus* sunny localities in Frankfurt am Main

The experimental units were exposed to 15 °C, 20 °C and 25 °C at a 16:8 h light:dark photocycle in environmental chambers (MKKL 1200, Flohr Instruments GmbH, Utrecht, The Netherlands). These experimental temperatures had been derived from *in situ* measurements in potential mosquito microhabitats (Fig. 1).

#### Larval food supply

The larvae were fed with animal-based fish food (Tetra Min®, Tetra, Melle, Germany) using a total amount of either 3 mg larva^-1^ or 6 mg larva^-1^. Larval food was provided on days 0, 3, 6, 9, 12, 15, 18 and 21 in the 15 °C treatments, on days 0, 2, 4, 6, 8, 10, 12 and 14 in the 20 °C treatments, and on days 0, 2, 4, 5, 7, 8, 9 and 10 in the 25 °C treatments. Food portions of either 0.25 or 0.5 mg larva^-1^ were offered during the first to fourth feeding and 0.5 or 1 mg larva^-1^ at later ones. A total food quantity of 3 mg larva^-1^ was considered as limited (at least at 20 °C) and 6 mg larva^-1^ as adequate for *Ae. albopictus* larval development (at least at 20 °C and 25 °C; see Müller et al. [38]). The feeding protocol also allowed for an optimal development of *Cx. pipiens molestus* at 20 °C and 25 °C as shown by Kreß et al. [33].

#### Effect variables

Mortality, pupal development time and pupal growth parameters (size, weight) were examined. During the daily census, pupae were collected and preserved in 70% ethanol. Pupal abdominal length (AL, 3^rd^ to 8^th^ abdominal segment) was measured using a stereo microscope and the software DISKUS (Carl H. Hilgers, Königswinter, Germany) within 14 days after collection. The gender of the pupae was determined by examination of their genital lobes. Species identity was determined using 4 criteria because overlapping morphological traits were occasionally observed: (i) the colour of the prothoracic trumpets (respiratory tubes); (ii) general pigmentation; (iii) paddle marginal spicule hairs; and (iv) compaction of the habitus. After gender and species identification, individual pupae were dried at 60 °C for 14 ± 2 h, and their dry weight (DW) was measured using a microbalance (± 0.01 μg, Sartorius model 708501, Göttingen, Germany).

#### Physicochemistry in test vessels at 15 °C

Physicochemical effects on larval ratio, temperature and food regime were investigated in the medium of test vessels kept at 15 °C due to long algal growth. After the last pupation had occurred in a given replicate, the following parameters were quantified (in parentheses: test used): standing time of vessel (days until last pupation occurred), concentrations of chlorophyll *a* (algal cells > 0.45 μm; guideline DIN 38 412 [39]), silicon dioxide (LCW028 test, Hach-Lange, Düsseldorf, Germany), nitrite (Aquaquant/Aquamerck tests, Merck, Darmstadt, Germany), nitrate (LCK 339, Hach-Lange), ammonium (Aquaquant/Aquamerck, Merck), phosphate (Aquamerck, Merck), pH and conductivity (TetraCon 325 and SenTix, WTW, Weilheim, Germany).

In every environmental chamber used in this study, there had previously been some vessels with *Scenedesmus obliquus* cultures and outdoor mesocosm water which probably led to an aerial proliferation of algal cells. Therefore, 10 ml aliquots of test media kept at 15 °C were diluted (1:10) with tap water, and phytoplankton were collected for 3 h in Utermöhl chambers. The number of algal cells per ml and replicate was estimated based on the microscopic counting of 2 random fields of 116.28 mm^2^ (Axiovert 40 C microscope, ZEISS; DISKUS software, Carl H. Hilgers, Königswinter). Algae were identified to family or genus level according to Streble et al. [40] and Linne von Berg [41].

### Data analysis

Data processing, graphing and statistics were performed using Microsoft Excel, GraphPad Prism version 7 and Statistica version 8 software. Effect variables and physicochemical measures are reported as mean ± standard deviation unless stated otherwise.

#### Data processing

Means of species- and gender-specific DWs and ALs were tabulated per replicate. Also, species-specific mortalities (%), gender-specific averages of the mean pupation time (PT_50_) and gender-specific daily biomass accumulation rates (BA) were calculated for every replicate. The PT_50_ indicated the time when 50% of male or female larvae had metamorphosed to pupae (for detailed calculations see Müller et al. [38]). The BA [mg larva^-1^ day^-1^] was calculated as the average for each gender and replicate by the division of the root of the mean DW and the root of the PT_50_. It should be noted that the BA neglected the initial DW of first-instar larvae.

Treatment-specific relative crowding coefficient values (RCC) were calculated with the AL, BA, DW and PT_50_ of female and male *Ae. albopictus* (RCC_Ae_) and *Cx. pipiens molestus* (RCC_Cx_) from the means of 4 replicates as described by Harper [42] and modified by Novak et al. [13] and Oberg et al. [34]: RCC_species A_ = {[0.5 × (species A^20:10^/ species B^20:10^) + (species A^15:15^/ species B^15:15^) + 2 × (species A^10:20^/ species B^10:20^)]/3} / {species A^30:00^/ species B^30:00^}. From these parameter-specific RCCs, the difference between species was calculated for each gender and experimental group to improve the comparability of parameter-specific competitiveness between 2 species [43]. Also, integrated RCCs for every gender and species and every experimental group were calculated from 3 parameter-specific RCCs [DW-RCC, AL-RCC, PT_50_-RCC; mean ± SD].

#### Statistics

The mosquito and physicochemical datasets were tested for Gaussian distribution using the Kolmogorov-Smirnov test and the D’Agostino Pearson omnibus normality test (*P* ≤ 0.01). Variance homogeneity of the data was tested using the Cochran’s test at the level of *P* ≤ 0.01. After confirmation of variance homogeneity for arcsin and log-transformed mortality [%], log-transformed PT_50_ data (gender-specific) and parameter-specific RCC values, four-factorial ANOVAs (independent factors: species, larval*,* temperature, food regime) and subsequent Tukey HSD tests were computed for these response variables. The heteroscedastic, but normally distributed dependent variables AL, BA and DW were analyzed with general linearized normal-log models with backward factor removal during model building and subsequent Wald statistics. The total average mortality of the two species (irrespective of experimental treatment) was compared with a t-test.

For reasons of straightforward whole-plot interpretation, polynomic regressions of second order (least squares fit) were computed with mortality data against larval ratio. Also, first order regressions (least squares fit) were computed with AL, DW and BA data against the number of larvae of the other species, respectively. Extra-sum-of-squares-tests were applied to compare independent fits with global fits for mosquito responses to food regime within a temperature block (mortality, corrected by Benjamini-Hochberg (BH) procedure with false discovery rate (FDR) of 0.05) and independent fits with global fits for each species and gender and within each temperature (AL, BA, BH corrected with FDR of 0.05), respectively.

Water quality parameters examined at 15 °C were tested by 2-way ANOVA (*P* < 0.05) for their dependence on initial larval ratio and food. Water quality parameters were further tested for correlation with algal growth (Spearman). The dependence of algal growth at 15 °C from initial larval ratio or larval mortality and food regime and their interactions were tested with 2-way ANOVAs. The algal growth data at 15 °C were tested for Spearman's correlation with *Ae. albopictus* mortality at 15 °C and *Cx. pipiens* mortality at 15 °C, respectively. Also, non-parametric Spearman's correlation analyses with the initial larval quantity of *Ae. albopictus* or *Cx. pipiens molestus* and the cell number of specific algal groups were performed for both food-related subplots.

## Results

### Larval mosquito habitats

During the field survey in the Rhine-Main metropolitan region, 170 intermittent and 8 permanent water bodies were identified as mosquito microhabitats. In total, 1072 mosquito larvae (606 *Cx. pipiens* (*s.l*.) (see Fig. 1a), 400 *Culex torrentium*, 3 *Culex hortensis*, 1 *Culex territans*, 37 *Culiseta annulata*, 1 *Aedes geniculata*, 3 *Anopheles maculipennis*, and 12 *Anopheles plumbeus*) were collected. The physicochemical parameters of a subset of *Cx. pipiens* (*s.l*.) positive microhabitats (*n* = 70) of mainly anthropogenic origin (88.6%) averaged to 15.0 ± 4.56 °C, 6.74 ± 4.02 mg l^-1^ dissolved oxygen, 390 ± 574 μS cm^-1^ electrical conductivity and pH 7.60 ± 0.84 (Fig. 1a).

### Outdoor versus experimental temperature

From mid-May to the end of July 2011, the water temperature in 1 l plastic cups was on average 19.2 ± 1.8 °C at the shaded site and 20.6 ± 4.8 °C at the sunny site (Fig. 1b). Minimum daily average temperatures were 14.2 °C and 14.1 °C in the shaded and sun-exposed test vessel, and maximum daily average temperatures, 25.3 °C and 27.9 °C, respectively. Thus, the temperatures chosen for the replacement series experiment (15, 20 and 25 °C) mirrored the minimum, mean and maximum daily average water temperatures in potential mosquito microhabitats during spring and summer in Frankfurt am Main, Germany.

### Multifactorial impact on mosquito response

#### General factorial response

The single factors species and temperature influenced every tested effect variable significantly (ANOVA, Table 1). This was analogous to the factor larval food regime except its non-significance for PT_50_ and male BA. In contrast, the single factor larval ratio had a significant influence on mortality and PT_50_ only. At the level of two-factorial interactions, food × temperature was always significant and species × food and species × temperature almost always significant for mosquito response variables (16 out of 18 sub-models). In contrast, the larval ratio in combination with species, food or temperature was significant in only 8 out of 27 sub-models. Three-factorial interactions of species × food × larval ratio strongly influenced the data (except AL and male DW), as did species × food × temperature (except BA). The triple interaction species × larval ratio × temperature had a significant influence on mortality, male PT_50_ and AL, whereas food × larval ratio × temperature had no significant influence on any effect variable. The four-factorial interaction was significant for female BA, female PT_50_ and male DW only (Table 1).

**Table 1:** Results for four-factorial significance tests (*df*, degree of freedom) with the independent factors species (*Aedes albopictus*, *Culex pipiens*), food regime (3 and 6 mg larva^-1^ food supply), larval ratio (0, 10, 15 and 20 larvae of the other species) and temperature (15, 20 and 25 °C). The dependent variables mortality (arcsin log-transformed) and female/male PT_50_ (log-transformed) were tested with a general linear model (ANOVA, *F* value) whereas the effect variables female/male abdominal length (AL), dry weight (DW) and biomass accumulation (BA) were tested with a general linearized model (normal-log model with backward removal, Wald value)

		Mortality		Female PT_50_		Male PT_50_		Female AL		Male AL		Female DW		Male DW		Female BA		Male BA	
	*df*	*F*	*P*	*F*	*P*	*F*	*P*	Wald	*P*	Wald	*P*	Wald	*P*	Wald	*P*	Wald	*P*	Wald	*P*
Intercept	1	1082	<0.001	130948	<0.001	117040	<0.001	83928	<0.001	67157	<0.001	3431	<0.001	21209	<0.001	49678	<0.001	51860	<0.001
Species (S)	1	5.66	0.019	64.00	<0.001	94.01	<0.001	105.6	<0.001	10.58	0.001	562.6	<0.001	1247	<0.001	58.61	<0.001	76.22	<0.001
Food (F)	1	6.26	0.014	3.18	0.076	1.65	0.201	87.39	<0.001	15.03	<0.001	48.55	<0.001	6.20	0.013	4.99	0.026	0.15	0.699
Larval ratio (L)	3	5.20	0.002	3.55	0.016	2.55	0.058	3.23	0.358	3.66	0.301	11.36	0.010	3.75	0.289	7.24	0.065	1.39	0.708
Temperature (T)	2	39.44	<0.001	1839	<0.001	1823	<0.001	71.84	<0.001	152.74	<0.001	127.87	<0.001	79.36	<0.001	1092	<0.001	787.2	<0.001
S × F	1	4.91	0.029	0.88	0.350	15.09	<0.001	53.44	<0.001	8.53	0.003	31.08	<0.001	35.89	<0.001	11.98	0.001	17.38	<0.001
S × L	3	9.89	<0.001	0.82	0.486	1.29	0.280	7.60	0.055	4.41	0.220	14.20	0.003	13.64	0.003	3.43	0.330	3.81	0.283
F × L	3	1.04	0.377	2.34	0.076	2.34	0.076	17.62	0.001	7.57	0.056	2.67	0.445	9.20	0.027	0.44	0.932	2.01	0.570
S × T	2	0.83	0.436	54.59	<0.001	47.60	<0.001	83.33	<0.001	45.81	<0.001	70.79	<0.001	57.32	<0.001	65.30	<0.001	38.20	<0.001
F × T	2	16.80	<0.001	25.62	<0.001	25.95	<0.001	16.87	<0.001	32.41	<0.001	76.47	<0.001	73.05	<0.001	40.15	<0.001	17.68	<0.001
L × T	6	1.78	0.108	0.77	0.596	0.28	0.945	16.47	0.011	15.32	0.018	6.32	0.388	14.85	0.021	10.61	0.101	5.46	0.486
S × F × L	3	3.45	0.019	5.24	0.002	7.98	<0.001	0.75	0.861	7.66	0.053	10.92	0.012	6.41	0.093	11.04	0.011	9.35	0.025
S × F × T	2	7.58	0.001	11.66	<0.001	6.07	0.003	10.04	0.007	37.00	<0.001	46.56	<0.001	38.93	<0.001	0.47	0.791	0.99	0.610
S × L × T	6	3.32	0.005	1.76	0.112	2.33	0.036	27.13	<0.001	14.08	0.029	6.25	0.395	9.86	0.131	5.26	0.511	8.18	0.225
F × L × T	6	0.63	0.709	0.85	0.532	0.77	0.594	4.29	0.637	5.29	0.507	9.00	0.174	12.33	0.055	9.99	0.125	0.93	0.988
S × F × L × T	6	0.67	0.670	2.94	0.010	1.21	0.307	5.05	0.537	4.40	0.622	3.52	0.741	15.07	0.020	15.45	0.017	6.45	0.375

#### Mortality

**Fig. 2:**
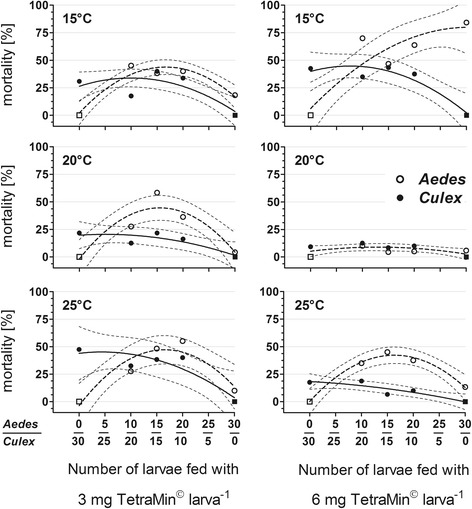
Larval mortality [mean, SD] of *Aedes albopictus* and *Culex pipiens* in pure and mixed cohorts (x-axis - the number of *Ae. albopictus*: *Cx. pipiens* larvae) in dependence of temperature and larval food regime. Squares symbolize artificially set zero values; solid lines and broken lines - non-linear regression (second-order model); dotted lines - 95% confidence interval

The split-plot design used in the 4-factorial replacement series experiment resulted in a larval mortality within the margins of expectation for intra- and interspecific competition with a larval density of 50 larvae l^-1^ (Fig. 2). The total average mortality (mean ± standard error of the mean; CV - coefficient of variation; *n* = 96) of *Ae. albopictus* (34.9 ± 2.67%; 74.8%) was significantly higher than the total average mortality of *Cx. pipiens molestus* (25.2 ± 1.94%; 75.4%; *t*_(95)_ = 13.0, *P* < 0.0001). The highest mortality of *Ae. albopictus* (66.2 ± 6.04%) and *Cx. pipiens molestus* (39.6 ± 4.39%) was observed at 15 °C and 6 mg larva^-1^ food supply. The *Cx. pipiens molestus* larvae had an equally high mortality (39.6 ± 6.14%) at 25 °C and 3 mg larva^-1^ food supply. The lowest mortality of *Ae. albopictus* was 8.54 ± 2.46% and that of *Cx. pipiens molestus* 10.0 ± 2.17%, both in cohorts exposed to 20 °C and supplied with 6 mg larva^-1^ food.

Mortality was mainly influenced by temperature. Further variance was produced by the factors food, species and larval ratio, by the 2-factorial interactions food × temperature, species × larval ratio, species × food, and the 3-factorial interactions species × food × temperature, species × food × larval ratio and species × larval ratio × temperature (Table 1). Between species, regression lines did differ slightly among cohorts fed 6 mg larva^-1^ and exposed to 20 °C (*F*_(3.34)_ = 1.87, BH-corrected *P* = 0.05), and those fed 3 mg larva^-1^ at 25 °C (*F*_(3.34)_ = 3.78, BH-corrected *P* = 0.042). In the other 4 temperature-food treatments, the mortality in pure and mixed cohorts differed strongly between species (15 °C, 3 mg larva^-1^: *F*_(3.34)_ = 5.86, BH-corrected *P* = 0.025; 15 °C, 6 mg larva^-1^: *F*_(3.34)_ = 3.78, BH-corrected *P* = 0.008; 20 °C, 3 mg larva^-1^: *F*_(3.34)_ = 3.78, BH-corrected *P* = 0.033; 25 °C, 6 mg larva^-1^: *F*_(3.34)_ = 3.78, BH-corrected *P* < 0.017; Fig. 2).

The presence of *Cx. pipiens molestus* larvae significantly increased the mortality of *Ae. albopictus* in the cohorts fed 6 mg larva^-1^ at 25 °C (Ae:Cx^15:15^ *vs* Ae:Cx^30:00^, Tukey HSD MS_(126)_ = 0.55, *P* = 0.046) and 3 mg larva^-1^ at 20 °C (Ae:Cx^15:15^ *vs* Ae:Cx^30:00^, Tukey HSD MS_(126)_ = 0.55, *P* = 0.023) and 25 °C (Ae:Cx^15:15^ *vs* Ae:Cx^30:00^, Tukey HSD MS_(126)_ = 0.55, *P* = 0.009; Ae:Cx^20:10^ *vs* Ae:Cx^30:00^, Tukey HSD MS_(126)_ = 0.55, *P* = 0.002). The species × food × larval ratio interaction on mortality was demonstrated by several significant differences between food-related *Ae. albopictus* cohorts: The Ae:Cx^15:15^ cohort that had been fed 3 mg larva^-1^ differed from the cohorts Ae:Cx^10:20^, Ae:Cx^20:10^ and Ae:Cx^30:00^ provided with 6 mg food larva^-1^ at 20 °C (Tukey HSD MS_(126)_ = 0.55, *P* = 0.004, *P* = 0.039, *P* = 0.001, respectively); the Ae:Cx^15:15^ cohort fed 3 mg larva^-1^ from the Ae:Cx ^30:00^ cohort fed 6 mg larva^-1^ at 25 °C (Tukey HSD MS_(126)_ = 0.55, *P* = 0.039), and the Ae:Cx^30:00^ cohort fed 3 mg larva^-1^ from the Ae:Cx^15:15^ cohort fed 6 mg larva^-1^ at 25 °C (Tukey HSD MS_(126)_ = 0.55, *P* = 0.010). In contrast, the presence of *Ae. albopictus* larvae did not influence the mortality of *Cx. pipiens molestus* (Tukey HSD MS_(126)_ = 0.55, *P* > 0.05).

#### Mean pupation time (PT_50_)

Species and temperature were the major factors affecting the PT_50_ of male and female larvae (Table 1). Also, a larval ratio significantly influenced the PT_50_ of female larvae while the single factor food had no significant effect (*P* > 0.08). The PT_50_ of male and female larvae was additionally influenced by the 2-factorial interactions species × temperature, food × temperature and species × food and the 3-factorial interactions species × food × larval ratio and species × food × temperature. The triple interaction species × larval ratio × temperature had a significant effect on the PT_50_ of male, and the 4-factorial combination on that of female larvae only_._

**Fig. 3:**
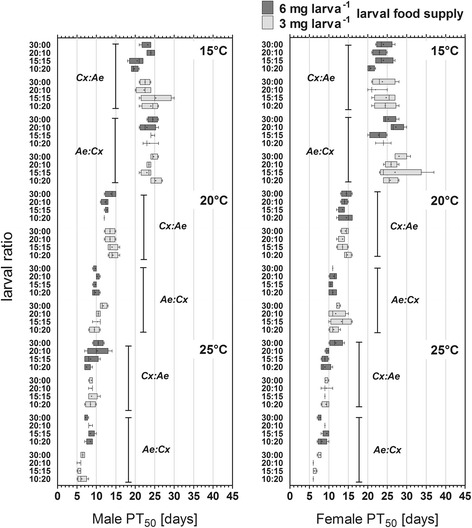
Mean pupation time PT_50_ (days; box-plot with Tukey whiskers, mean shown as ‘+’) of *Aedes albopictus* and *Culex pipiens* in dependence of gender, the larval ratio (number of *Ae. albopictus*: *Cx. pipiens* larvae - Ae: Cx), temperature and larval food regime

The PT_50_ of both genders and species decreased as a function of temperature, with maxima at 15 °C and minima at 25 °C (Fig. 3). At 25 °C, a food supply of 3 mg larva^-1^ decreased the mean PT_50_ of females by 2.1 days (*Ae. albopictus*) and 0.7 days (*Cx. pipiens molestus*) and the mean PT_50_ of males by 2.1 days (*Ae. albopictus*) and 0.5 days (*Cx. pipiens molestus*) if compared to the cohorts fed 6 mg larva^-1^ at 25 °C. At lower temperatures, the PT_50_ had an opposite trend with a higher average PT_50_ of females of 1.9 days (*Ae. albopictus*) and 0.8 days (*Cx. pipiens molestus*) at 15 °C and 1.2 days (*Ae. albopictus*) at 20 °C if larvae had been supplied with 3 mg food larva^-1^, although the average PT_50_ of *Cx. pipiens molestus* was similar in both food treatments at 20 °C.

This general pattern of 2- and 3-factorial interactions of species × food × temperature equally applied to the average PT_50_ of male larvae that had received 6 mg food larva^-1^, which was 0.3 days (*Ae. albopictus*) and 1.6 days (*Cx. pipiens molestus*) shorter at 15 °C and 0.7 days (*Ae. albopictus*) and 1.1 days (*Cx. pipiens molestus*) shorter at 20 °C than the average PT_50_ of males that had been fed with 3 mg larva^-1^. With regard to larval ratio, the PT_50_ of females decreased with an increasing larval ratio of the competing species, but this was only observed in the cohorts of *Ae. albopictus* provided with 3 mg food larva^-1^ and those of *Cx. pipiens molestus* given 6 mg larva^-1^.

#### Pupal growth

Pupal growth (AL, DW) was mainly influenced by the single factors species, temperature and food and their 2- and 3-factorial interactions (Table 1). The larval ratio had a significant effect only if it interacted with the factors species (DW), food (AL of females, DW of males) and temperature (AL, DW of males) or in concert with species × food (DW of females) and species × temperature (AL). In the following, the detailed response patterns are described exclusively for the pupal growth of females due to their biological significance for size/weight-fecundity-relationships [21]. However, male response patterns were similar.

The AL of female *Ae. albopictus* was generally greater (3.12 mm) than that of female *Cx. pipiens molestus* (2.89 mm), although it was equal at 20 °C and a food supply of 6 mg larva^-1^ (*F*_(2.28)_ = 1.25, *P* = 0.30), and at 15 °C and 3 mg food larva^-1^ (*F*_(2.27)_ = 3.14, *P* = 0.06) (Additional file 2: Figure S2). The greatest AL of female *Ae. albopictus* was measured in the cohort fed with 3 mg larva^-1^ at 20 °C, followed by the cohort given 6 mg larva^-1^ at 20 °C, the significantly different larval food regimes at 25 °C (6 mg larva^-1^: 3.26 ± 0.15 mm; 3 mg larva^-1^: 2.99 ± 0.12 mm, *F*_(2.26)_ = 16.01, *P* < 0.0001) and finally by both larval food regimes at 15 °C.

The AL of female *Cx. pipiens molestus* differed significantly among the larval food regimes at every tested temperature (15 °C: *F*_(2.27)_ = 17.26, *P* = 0.0001; 20 °C: *F*_(2.28)_ = 12.25, *P* = 0.0002; 25 °C: *F*_(2.27)_ = 23.39, *P* = 0.0002). The maximum AL of female *Cx. pipiens molestus* was measured in the cohort with 6 mg food larva^-1^ at 15 °C, followed by the groups fed 6 mg larva^-1^ and 3 mg larva^-1^ at 20 °C, the 3 mg larva^-1^ cohort at 15 °C, and finally by both larval food regimes at 25 °C.

The mean DW of female *Cx. pipiens molestus* (0.80 mg) was higher than that of female *Ae. albopictus* (0.58 mg). In both species, the DW of females was always largest in the cohorts with 6 mg larva^-1^ food supply (Additional file 3: Figure S3). In the cohorts fed 6 mg larva^-1^, the DW of *Ae. albopictus* increased with increasing temperature, whereas the DW of *Cx. pipiens molestus* decreased with increasing temperature. In the cohorts provided with 3 mg food larva^-1^, there was no temperature-dependent change of DW. By overall comparison, species-specific differences in DW were minor at 25 °C under both food regimes. In the *Cx. pipiens molestus* cohorts given 6 mg food larva^-1^ at 15 °C and 20 °C, a decreased DW became apparent as heterospecific larval numbers increased, but this was not the case at 25 °C nor for *Ae. albopictus* (Additional file 3: Figure S3).

#### Daily increase in weight (BA)

The daily increase of weight (BA) was mainly influenced by the factors species (mean BA_*Ae*_ = 0.053 mg larva^-1^ day, mean BA_*Cx*_ = 0.058 mg larva^-1^ day) and temperature (Table 1). Minimum and maximum values of BA were always found at 15 °C and 25 °C (cohorts fed 3 mg larva^-1^), demonstrating the strong impact of temperature on BA. The single factor food had a significant influence on females only, but food × species or food × temperature and the 3-factorial interaction species × food × temperature had significant effects on both males and females. The BA of both species was similar to 3 mg larva^-1^ food supply except *Ae. albopictus* at 25 °C, but the BA was lower in *Ae. albopictus* than *Cx. pipiens molestus* cohorts fed with 6 mg larva^-1^.

**Fig. 4:**
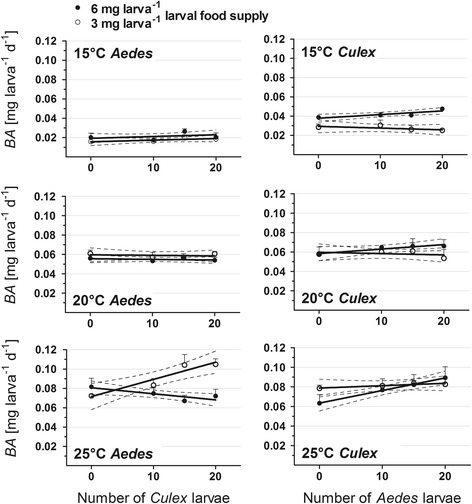
Daily accumulation of biomass (*BA*, mean ± SD) of individual female *Aedes albopictus* and *Culex pipiens* during their aquatic part of life in dependence of larval ratio, temperature and larval food regime. Solid line - linear regression; dotted lines - 95% confidence interval

Similar to AL, the BA of *Ae. albopictus* fed 6 mg larva^-1^ at 25 °C slightly decreased with an increasing number of *Cx. pipiens molestus* larvae, whereas the BA of those fed 3 mg larva^-1^ increased with an increasing number of *Cx. pipiens molestus* larvae (Fig. 4). In contrast, the BA of *Cx. pipiens molestus* did not change with different heterospecific larval ratios. This complex pattern led to larval ratio having a significant influence only within the combination species × food × larval ratio or within the 4-factorial interaction (Table 1).

#### Relative crowding coefficient (RCC)

Both species exhibited a weak to intermediate interspecific competitiveness with regard to growth and development as indicated by RCC values between 0.91 and 2.31 (Table 2; Additional file 4: Table S1; [18, 43]). Relative crowding coefficient values < 1.39 were observed in 38 out of 96 experimental groups indicating no competition between species in 39.6% of treatments (Additional file 4: Table S1). Values between 1.4 to 2.31 indicative of low to intermediate competition were most frequently measured (56.3% for RCC_Ae_, 58.3% RCC_Cx_), whereas RCC values ≥ 2.5 signifying high competition were never observed.

**Table 2:** Relative crowding coefficients (RCCs). Integrated relative crowding coefficient values for female and male *Aedes albopictus* and *Culex pipiens* in different experimental treatments indicate a generally low competition between species regarding growth and development due to equilibration of the traits of different species. To evaluate the predomination of one species trait over the other in more detail, the differences between RCC_Ae_ and RCC_Cx_ that are based on biomass accumulation rate (BA), pupal dry weight (DW), pupal abdominal length (AL) and mean pupation time (PT_50_) are additionally reported, whereas the respective parameter-specific RCCs can be found in the Additional file 4: Table S1

Larval exposure		Integrated RCC_Ae_ (mean ± SD)	Integrated RCC_Cx_ (mean ± SD)	AL-RCC_Ae_ - AL-RCC_Cx_^a^	DW-RCC_Ae_ - DW-RCC_Cx_^a^	PT_50_-RCC_Ae_ - PT_50_-RCC_Cx_^a^	BA-RCC_Ae_ - BA-RCC_Cx_^a^
6 mg larva^-1^	15 °C	1.57 ± 0.07	1.34 ± 0.06	0.38	0.16	0.15	-0.29
	20 °C	1.52 ± 0.15	1.38 ± 0.12	-0.04	0.46	0.03	-0.50
	25 °C	1.67 ± 0.35	1.28 ± 0.24	-0.03	0.13	1.07	-1.35
3 mg larva^-1^	15 °C	1.40 ± 0.19	1.51 ± 0.19	0.31	-0.38	-0.28	0.75
	20 °C	1.45 ± 0.06	1.45 ± 0.07	0.04	0.11	-0.15	0.05
	25 °C	1.28 ± 0.13	1.64 ± 0.17	-0.18	-0.20	-0.70	0.88
6 mg larva^-1^	15 °C	1.59 ± 0.14	1.32 ± 0.12	0.00	0.50	0.32	-0.84
	20 °C	1.52 ± 0.19	1.39 ± 0.18	-0.29	0.33	0.36	-0.66
	25 °C	1.72 ± 0.46	1.27 ± 0.29	-0.07	0.11	1.32	-1.40
3 mg larva^-1^	15 °C	1.38 ± 0.11	1.52 ± 0.11	0.12	-0.30	-0.22	0.50
	20 °C	1.42 ± 0.36	1.53 ± 0.35	-0.32	0.68	-0.67	0.02
	25 °C	1.40 ± 0.02	1.49 ± 0.02	-0.08	-0.06	-0.13	0.19

^a^A negative value indicates the superiority of a *Cx. pipiens* trait and positive value the superiority of an *Ae. albopictus* trait

The pattern of interspecific competitiveness described by parameter-specific RCCs was similar between genders but altered by larval exposure conditions and the parameter. The number of groups with the superiority of *Ae. albopictus* or *Cx. pipiens molestus* (groups with negative *versus* positive parameter-specific RCC differences) was balanced (Table 2). Disregarding temperature, PT_50_ and BA produced a mirrored RCC difference pattern with the dominance of the PT_50_-RCC and a parallel lesser BA-RCC in the *Ae. albopictus* cohorts fed 6 mg larva^-1^ and the *Cx. pipiens molestus* cohorts given 3 mg larva^-1^, respectively. The DW-RCC of *Ae. albopictus* was larger in the *Ae. albopictus* cohorts fed 6 mg larva^-1^ and those fed 3 mg larva^-1^ at 20 °C. The AL-RCC was higher in *Ae. albopictus* than in *Cx. pipiens molestus* cohorts at 15 °C, but the AL-RCC of *Cx. pipiens molestus* became higher than in *Ae. albopictus* at a higher temperature.

The integration of RCCs revealed that differences from growth and development specific RCCs were almost balanced out given that the single factors species, gender, food and temperature and almost all interactions had no significant influence. Notably, the integrated RCC_Ae_ was higher in the 6 mg larva^-1^ food treatment and the integrated RCC_Cx_ in the 3 mg larva^-1^ food treatment (species × food interaction, *P* < 0.001).

### Water parameters in test media of 15 °C treatments

The total standing time of test vessels in parallel with the maximum pupation time was altered by larval ratio and larval food regime: The longest standing time of 40 ± 0 days was observed in the Ae:Cx^00:30^ cohort provided with 6 food mg larva^-1^and the shortest standing time of 33 ± 2 days in the Ae:Cx^00:30^ cohort fed with 3 mg larva^-1^ (Table 3). Physicochemical water parameters depended significantly on the larval food regime (ammonium, nitrate, nitrite, silicon dioxide, oxygen, pH), the larval ratio (nitrate) and their interaction (silicon dioxide). At the end of the half-life-cycle tests where larvae had been fed with 6 mg larva^-1^, the concentrations of nitrate (2.4 to 6-fold) and potentially toxic nitrite (2-fold) were lower than in the water of 3 mg larva^-1^ cohorts. In contrast, concentrations of ammonium (1.7 to 18-fold), silicon dioxide (1.8 to 5.6-fold; apart from 0.45-fold in the Ae:Cx^30:00^ treatment), oxygen (1.1 to 1.3-fold) and pH (1.2 to 1.3-fold) were higher than in the water of cohorts fed with 3 mg larva^-1^ (Table 3).

**Table 3:** Physicochemistry of test media at 15 °C. Chemical, physical and biological parameters (mean ± SD) and standing time (d, mean ± SD) of test vessels during the half life-cycle experiment with *Aedes albopictus* and *Culex pipiens* based on the larval ratio (number of *A. albopictus*: *C. pipiens* larvae; Ae:Cx) and food quantity (3 or 6 mg larva^-1^ food supply)

Ae:Cx	NO_2_	NO_3_	NH_4_	PO_4_	pH	Con	O_2_	Chl *a*	SiO_2_	ST
6 mg larva^-1^ food supply										
00:30	0.01 ± 0.00	1.16 ± 0.15	0.88 ± 0.25	2.50 ± 0.58	9.92 ± 0.24	271.5 ± 10.79	15.02 ± 2.99	0.42 ± 0.28	4.53 ± 1.00	40 ± 0
10:20	0.01 ± 0.00	2.72 ± 2.33	0.75 ± 0.29	2.50 ± 0.91	9.75 ± 0.36	297.0 ± 16.02	15.75 ± 1.89	0.24 ± 0.12	1.90 ± 1.54	36 ± 4
15:15	0.01 ± 0.00	0.93 ± 0.52	0.75 ± 0.29	2.50 ± 1.29	9.98 ± 0.13	271.3 ± 16.21	18.18 ± 0.84	0.12 ± 0.03	4.10 ± 2.19	35 ± 5
20:10	0.01 ± 0.00	0.94 ± 0.22	0.75 ± 0.29	3.13 ± 0.85	9.86 ± 0.30	269.7 ± 42.62	17.12 ± 2.30	0.39 ± 0.34	2.70 ± 1.90	38 ± 3
30:00	0.01 ± 0.01	0.84 ± 0.22	2.00 ± 2.0	4.00 ± 1.41	9.78 ± 0.38	313.3 ± 34.73	14.41 ± 2.87	0.41 ± 0.09	0.54 ± 0.16	36 ± 3
3 mg larva^-1^ food supply										
00:30	0.02 ± 0.01	6.15 ± 0.91	0.05 ± 0.00	3.00 ± 0.00	7.97 ± 0.04	238.5 ± 16.05	13.25 ± 0.91	0.88 ± 0.58	0.81 ± 0.03	33 ± 2
10:20	0.02 ± 0.01	6.49 ± 0.97	0.05 ± 0.00	3.00 ± 0.00	7.91 ± 0.07	270.3 ± 55.45	13.05 ± 0.56	0.36 ± 0.15	1.05 ± 0.03	37 ± 1
15:15	0.02 ± 0.01	5.59 ± 0.60	0.23 ± 0.20	3.00 ± 0.00	7.53 ± 0.11	258.5 ± 13.99	13.67 ± 2.28	0.27 ± 0.19	0.92 ± 0.07	39 ± 1
20:10	0.02 ± 0.01	5.34 ± 0.73	0.45 ± 0.06	3.00 ± 0.00	7.97 ± 0.08	255.5 ± 12.47	13.50 ± 0.20	0.36 ± 0.22	0.91 ± 0.08	38 ± 1
30:00	0.02 ± 0.01	3.03 ± 0.71	0.45 ± 0.06	3.00 ± 0.00	8.18 ± 0.04	259.0 ± 23.76	12.99 ± 1.11	0.14 ± 0.05	1.20 ± 0.15	39 ± 1

*Abbreviations*: *NO*_*2*_^*-*^ nitrite (mg l^-1^), *NO*_*3*_^*-*^ nitrate (mg l^-1^), *NH*_*4*_^*+*^ ammonium (mg l^-1^), *PO*_*4*_^*2-*^ phosphate (mg l^-1^); pH (acidic or basic character), *Con* conductivity (μS cm^-2^), *O*_*2*_ oxygen content (mg l^-1^), *Chl a*, chlorophyll *a* (mg l^-1^), *SiO*_*2*_ silicon dioxide (mg l^-1^), *ST* standing time ≜maximum pupation time (d)

**Fig. 5:**
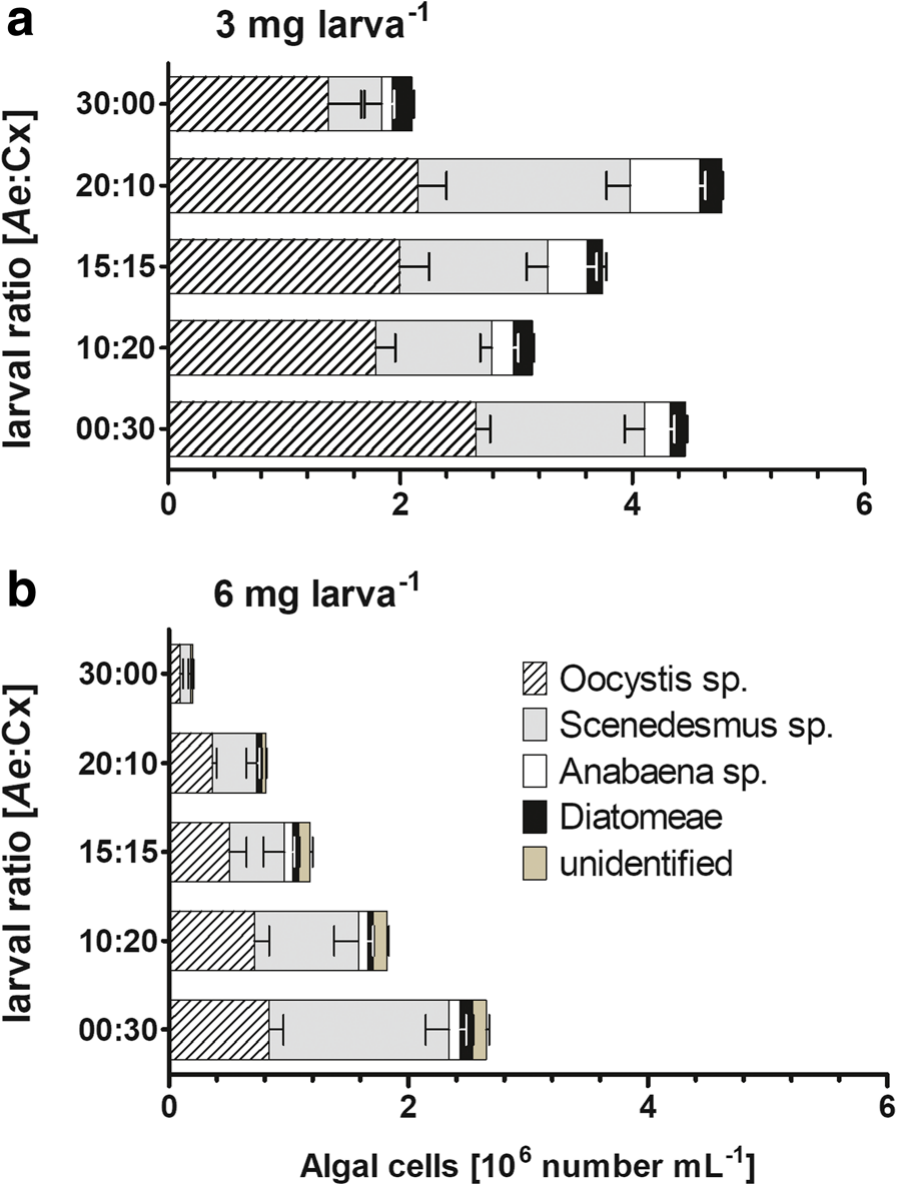
Algal growth in test vessels at 15 °C. Algal cells (mean and SD) grown in test media with pure and mixed cohorts of *Aedes albopictus* and *Culex pipiens* larvae in dependence of larval ratio (Ae:Cx - number of *Ae. albopictus*: *Cx. pipiens* larvae) and larval food regime with (**a**) 3 mg larva^-1^ larval food supply *versus* (**b**) 6 mg larva^-1^ larval food supply at 15 °C. Algal growth was not observed in test vessels at 20 °C and 25 °C

In correlation to increased oxygen (Spearman's *r*_(10)_ = -0.588, *P* = 0.04), conductivity (Spearman's *r*_(10)_ = -0.758, *P* = 0.006), pH (Spearman's *r*_(9)_ = -0.720, *P* = 0.014), ammonium (Spearman's *r*_(10)_ = -0.741, *P* = 0.007) and decreased nitrate (Spearman's *r*_(10)_ = 0.842, *P* = 0.001) and nitrite (Spearman's *r*_(10)_ = 0.801, *P* = 0.008), algal growth was significantly reduced in the water with cohorts fed 6 mg larva^-1^ (*F*_(1.50)_ = 116.3,, *P* < 0.001, 46.6% total variation) (Fig. 5). Notably, algal growth was negatively correlated to *Ae. albopictus* mortality (Spearman's *r*_(8)_ = -0.86, *P* = 0.011). Algal growth especially declined with an increasing number of initial *Ae. albopictus* larvae (larval ratio: *F*_(4.50)_ = 13.17, *P* < 0.001, 21.1% total variation; food × larval ratio: *F*_(4.50)_ = 4.58, *P* = 0.003, 7.3% total variation).

## Discussion

In the present study, we show that the physicochemical and microclimatic conditions of potential and actual mosquito breeding sites in Germany offer suitable conditions for a balanced coexistence and frequent competitive superiority of *Cx. pipiens molestus* over *Ae. albopictus* with regard to larval and pupal survival. We demonstrate that the interspecific competition pattern of these two mosquito species does not directly depend on the thermal regime (rejection of hypothesis i), but is rather controlled by food regime and species-food and species-temperature interactions (support of hypothesis ii). Furthermore, we discuss how the resource competition between *Ae. albopictus* and *Cx. pipiens molestus* is directly connected to species-specific foraging behaviour and physicochemical as well as phycological microhabitat parameters (support of hypothesis iii).

### Larval coexistence under present climatic conditions in Germany

In general agreement with our data, *Ae. albopictus* and *Cx. pipiens molestus* had an almost identical population growth at 20 °C but a divergent one at a higher temperature [33]. A simple extrapolation of the laboratory optimum to mosquito co-occurrence in the field suggests that microhabitats in Germany with a typical median temperature of 20 °C in spring and summer offer favourable conditions for the coexistence of these two species, even if the food regime may ultimately restrict the success of *Ae. albopictus* in mixed larval habitats. The longer development time of *Ae. albopictus* at 15 °C and *Cx. pipiens molestus* at 20 °C and 25 °C will directly translate to the population level [44] suggesting a shift from *Cx. pipiens* (*s.l*.) with advantaged population activity at 15 °C to *Ae. albopictus* at a higher temperature. During warmer seasons, *Ae. albopictus* may make up its population delay by faster development time, faster growth and superior biomass accumulation. This experimentally based expectation has support from field observations elsewhere [18, 27, 28]: In Italy, for example, the population growth of *Cx. pipiens* (*s.l*.) starts in mid-April and peaks in July, and that of *Ae. albopictus* in mid-May and September, respectively [18]. Marini et al. [28] calculated for *Ae. albopictus* an average delayed population growth by 29 days when compared with *Cx. pipiens* (*s.l*.) from the same location and year.

Our results indicate a general adaptation of *Cx. pipiens* to low temperatures although the thermal optimum of *Cx. pipiens* (*s.l*.) is between 20 °C and 25/27 °C (present study, [45, 46]). In contrast, the higher total average mortality of *Ae. albopictus* and the especially high mortality at 15 °C suggest that *Ae. albopictus* is less adapted to colder ecoregions than its autochthonous competitor. However, despite these differences in thermal biology, the outcomes from available competition studies for *Ae. albopictus* and *Cx. pipiens* (*s.l*.) might be transferrable to colder ecoregions [18, 27], because the temperature did not influence the interspecific pattern at other than optimal test conditions. We, therefore, reject hypothesis i, that a competitive superiority depends on the species-specific thermal tolerance spectrum and therefore *Ae. albopictus* might be advantaged at higher and *Cx. pipiens* (*s.l*.) at lower temperatures. Similarly, temperatures ranging from 24 to 30 °C did not change the larval competition between *Ae. albopictus* and *A. aegypti* [6], and a range of 15 °C to 31 °C did not alter the larval competition between *Ae. albopictus* and *O. triseriatus* [5].

### The competitive superiority of *Culex pipiens* over *Aedes albopictus* - or *vice versa*?

All available evidence from this study suggests a competitive superiority of *Cx. pipiens molestus* over *Ae. albopictus* with regard to survival at every other temperature and food treatment than 20 °C and 6 mg larva^-1^. Agonistic growth and development strategies levelled out the parameter-specific sublethal crowding effects. Mortalities comparable to the ones registered in the present study have been observed in *Cx. pipiens* (*s.l*.) cohorts regardless of the presence or absence of larvae of a second mosquito species, whereas the presence of *Cx. pipiens* larvae especially affected *Ae. albopictus* survival and biomass accumulation at 25 °C and 3 mg larva^-1^ food supply*.* Carrieri et al. [18] observed balanced survival and development in their *Ae. albopictus* and *Cx. pipiens* (*s.l*.) cohorts exposed to 25 °C with 2.85 mg larva^-1^ food supply, but growth differed significantly between species indicating a slightly suboptimal food supply especially for *Cx. pipiens* (*s.l*.). As an additional contrast to our study, the powerful competitive superiority of *Ae. albopictus* over *Cx. pipiens* (*s.l*.) became apparent at poorer larval food conditions at 20 °C and 25 °C [18]. Likewise, Costanzo et al. [27] reported competitive superiority of *Ae. albopictus* over *Cx. pipiens* (*s.l*.).

The contrary outcomes from the replacement series experiments in this study and those by Carrieri et al. [18] and Costanzo et al. [27] are not expected to result from the use of different *Ae. albopictus* mosquito strains [47, 48] but more likely from the investigation of different members of the *Cx. pipiens* complex with differential autecological characteristics [26]. The autogenic and stenogamic biotype *Cx. pipiens molestus* shows a homodynamic development, strong synanthropic behaviour, mammal feeding type and urban to suburban distribution [30, 49, 50]. This ecotype often occupies biotopes different from those of the ornithophilic *Cx. pipiens pipiens* in regions with cold temperate climate (hypogeous, [30]), although *Cx. pipiens molestus* is not excluded from epigeous breeding sites in the Rhine rift valley and warm temperate European regions [49, 51, 52].

However, resource competition may give a more reliable explanation for the opposite competition pattern observed between the present study and those by Carrieri et al. [18] and Costanzo et al. [27], because different types of food were supplied (animal-based food *versus* plant-based detritus), species-specific food quantity responses can be assumed [17, 18] and differential food quantity-temperature interactions may have taken place [5, 33, 38].

### Larval food supply is decisive for competitive superiority

Hypothesis ii stating food regime is a major determinant for interspecific interactions with temperature becomes supported by the comparison of our data with literature. In an overall comparison of three competition studies (present study, [18, 27]), the food supply in our experiment was perhaps always *ad libitum* as implied by evenly balanced biomass accumulation rates. On the other hand, pupal sizes and weights were always larger in the cohorts fed with 6 mg larva^-1^. This surplus of food may especially have favoured *Cx. pipiens molestus* in our study, whereas the progressively limited food resources in the studies by Carrieri et al. [18] and Costanzo et al. [27] may have resulted in a competitive superiority of *Ae. albopictus*. Similarly, *O. triseriatus* had a better survival than *Ae. albopictus* in water sampled from tree-holes with significant detritus whereas probably limited resource levels in tire water favoured *Ae. albopictus* [21]. In another replacement series experiment, the larval superiority of *Ae. albopictus* over *O. triseriatus* was observed under 0.5 mg but diminished under two mg larval food supply [5].

Food quality could have alternatively triggered the different competitive interactions between *Ae. albopictus* and *Cx. pipiens molestus* given that spatiotemporal natural variation in detritus quality (in terms of the animal to plant-based ratio and associated microbial growth) can shape mosquito assemblages [53]. It has been shown that the detritus type, its decay rate and associated microbial growth influence the interspecific competition between *Ae. albopictus* and *O. triseriatus* and *Ae. aegypti*, respectively [17, 53]. It has also been shown that *Ae. aegypti* is weaker in competition than *Ae. albopictus* if larvae feed on oak, pine, or insect detritus (related to low to intermediate microbial growth) but face competition if feeding on grass which is related to high microbial growth [17]. *Aedes albopictus* has been described as an opportunistic feeder [54] with great resistance to starvation [14], but animal-based detritus and associated microorganisms always yield higher performance of *Ae. albopictus* [20, 38, 53, 55, 56].

Under resource-limiting conditions (limiting at least for *Cx. pipiens molestus*), *Ae. albopictus* with its continuously foraging behaviour may usurp detritus, and specifically essential nutrients, more quickly than the selectively feeding larvae of *Cx. pipiens* (*s.l*.) which rest between meals [22, 56, 57]. *Culex pipiens molestus* larvae are known to react to phagostimulants released from high-value food which may allow the species to control foraging and filtering activity to usurp mostly food particles with high nutritional value [57, 58]. However, the opportunistic features of *Ae. albopictus* might not be successful in microhabitats with eutrophic conditions, as observed at 25 °C and 6 mg larva^-1^ food supply where the BA of *Ae. albopictus* even decreased, and the PT_50_ increased if compared to 3 mg larva^-1^ cohorts.

### Phycology is a crucial factor for *Ae. albopictus* survival

Our study demonstrates that resource competition between *Ae. albopictus* and *Cx. pipiens molestus* is directly connected to physicochemical and phycological parameters of their microhabitat (hypothesis iii). The competitive disadvantage of *Ae. albopictus* with lower survival under eutrophic conditions became very substantial in the cohorts fed 6 mg larva^-1^ at 15 °C. Here, the algal growth-mortality relationship directly pointed towards the importance of phycology in temperate mosquito ecology. Notably, *Ae. albopictus* ingests every available particle very quickly, especially green algal cells with an average of 22.4 ± 0.33 cells per second [59]. The green and blue-green algal growth in our study (*Oocystis*, *Scenedesmus*, *Anabaena*) were in fact negatively correlated to the initial *Ae. albopictus* larval ratio when 6 mg larva^-1^ food was supplied. Marten [22, 25] demonstrated a starvation effect of a variety of algal species, and in particular the lethal effect on first- and second-instars of *Ae. albopictus* if feeding on indigestible algal cells. For instance, larval feeding on *Scenedesmus obliquus* (the likely *Scenedesmus* species in our study) almost always killed *Ae. albopictus* first- and second-instar larvae, whereas *Ae. albopictus* first- and second-instar larvae survived feeding on some (but not every) species of *Scenedesmus*, *Oocystis* and *Anabaena* [25].

### Water physicochemistry suits both mosquito species

Water physicochemistry in the test vessels well resembled that of *Cx. pipiens* (*s.l*.) microhabitats at the northern border of the Rhine rift valley in Germany. The mosquito larvae had a direct impact on nutrient cycling (e.g. nitrate, silicon dioxide concentration) and algal growth, at least in our test vessels. According to physicochemistry, the ecology of *Cx. pipiens* (*s.l*.) is described as euryoecious [26] (β-mesosaprob microhabitats, [60]). For *Ae. albopictus*, however, the physicochemical parameters in the replacement series experiment were sometimes higher than reported in the few published studies in their natural microhabitats (pH = 7.97, dissolved oxygen ≤ 9.7 mg l^-1^, [23, 61]). A pH between 6.8 and 7.6 seems optimal, although *Ae. albopictus* larvae were observed within a pH range of 5.2 to 8.4 and at dissolved oxygen levels of 1.3 mg l^-1^ [60]. Wu & Chang [62] reported the greatest rate of food displacement of *Ae. albopictus* in water with a pH of 5.5. However, given that physicochemical conditions similar to our experiment were interference-free for *Ae. albopictus* larval/pupal performance at 20 °C to 30 °C [38], the physicochemistry of natural *Culex* spp. microhabitats in warmer regions of Germany, specifically in the Rhine-Main Metropolitan region, would not prevent an establishment of *Ae. albopictus*.

## Conclusions

Given recent distribution models [3, 4] and the fact that 67% of collected *Ae. albopictus* larvae shared their microhabitats with *Cx. pipiens* (*s.l*.) larvae in an Italian monitoring study [18], regular encounters between *Ae. albopictus* and *Cx. pipiens* (*s.l*.) larvae can be anticipated in suitable (container-like) temporary water bodies in Germany. Our results indicate that larval coexistence of *Cx. pipiens molestus* and *Ae. albopictus* is possible at present and forecasted future spring-to-autumn climate conditions of the Rhine rift valley, but competitive exclusion due to reduced larval survival of *Ae. albopictus* may be the more frequent condition in microhabitats where *Cx. pipiens* (*s.l*.) populations are established.

Next to the competitive superiority of *Cx. pipiens* (*s.l.*), the advantage of the faster development of *Cx. pipiens* (*s.l*.) at 15 °C may be of highest importance when available containers are initially colonized in spring and early summer, and to maintain population growth in autumn. However, the temperature was not a controlling factor for interspecific competition in our study, but food quantity, multifactorial interactions and food-related water parameter changes. Species-specific foraging behaviour influenced the physicochemistry and phycology in experimental mosquito microhabitats, and algal growth controlled especially the performance of *Ae. albopictus*. Thus, detailed examination of context dependence in interspecific interactions of these two important vector species and the related changes of water chemistry may lead to a better understanding of *Ae. albopictus* colonization in Germany [63] and elsewhere.

## Additional files

Additional file 1: Figure S1. Sampled localities positive for mosquito larvae in the Rhine-Main region. (PPTX 3726 kb)

Additional file 2: Figure S2. Pupal size. Abdominal length [AL, mm, mean ± SD] of female *Ae. albopictus* and *Cx. pipiens* in pure and mixed cohorts in dependence of temperature and larval food regime. Line - linear regression. Dotted lines - 95% confidence interval. (PPTX 558 kb)

Additional file 3: Figure S3. Pupal weight. Dry weight [DW, mg, mean ± 95% CI] of female *Ae. albopictus* and *Cx. pipiens* in pure and mixed cohorts in dependence of temperature and larval food regime. Line - linear regression. Dotted lines - 95% confidence interval. (PPTX 558 kb)

Additional file 4: Table S1. Parameter-specific relative crowding coefficients. Relative crowding coefficient values for female and male *Ae. albopictus* (RCC_Ae_) and *Cx. pipiens* (RCC_Cx_) based on biomass accumulation rate (BA), pupal dry weight (DW), pupal abdominal length (AL) and mean pupation time (PT_50_) are listed for the different experimental treatments. (DOCX 15 kb)

## Abbreviations

Ae: Cx^00:30^: Experimental units with only *Cx. pipiens molestus* larvae
Ae: Cx^10:20^: Asymmetrically mixed treatment with a 10:20 distribution of *Ae. albopictus*: *Cx. pipiens molestus* larvae
Ae: Cx^15:15^: Symmetrically mixed treatment with a 15:15 distribution of *Ae. albopictus*: *Cx. pipiens molestus* larvae
Ae: Cx^20:10^: Asymmetrically mixed treatment with a 20:10 distribution of *Ae. albopictus*: *Cx. pipiens molestus* larvae
Ae: Cx^30:00^: Experimental units with only *Ae. albopictus* larvae
AL: Abdominal length (mm)
AL-RCC: AL-specific relative crowding coefficient
ANOVA: Analysis of variance
BA: Gender-specific daily biomass accumulation rate (mg larva^-1^ day^-1^)
BA-RCC: BA-specific relative crowding coefficient
BH: Benjamini-Hochberg
*cox*1: Mitochondrial cytochrome *c* oxidase subunit 1
CV: Coefficient of variation
DW: Dry weight (mg)
DW-RCC: DW-specific relative crowding coefficient
FDR: False discovery rate
HSD: Honest significant difference
PT_50_: Gender-specific averages of the mean pupation time (days)
PT_50_-RCC: PT_50_-specific relative crowding coefficient
RCC: Relative crowding coefficient
RCC_Ae_: Integrated relative crowding coefficient for *Ae. albopictus*
RCC_Cx_: Integrated relative crowding coefficient for *Cx. pipiens molestus*
