# Supplementary material for: Larval superiority of Culex pipiens to Aedes albopictus in a replacement series experiment: prospects for coexistence in Germany
Source: Parasit Vectors. 2018 Feb 2;11:80. doi: 10.1186/s13071-018-2665-3 (PMC5797359; doi:10.1186/s13071-018-2665-3)
Supplement: Supplementary file 1 — Sampled localities positive for mosquito larvae in the Rhine-Main region. (PPTX 3726 kb) [file 13071_2018_2665_MOESM1_ESM.pptx]

## Slide 1
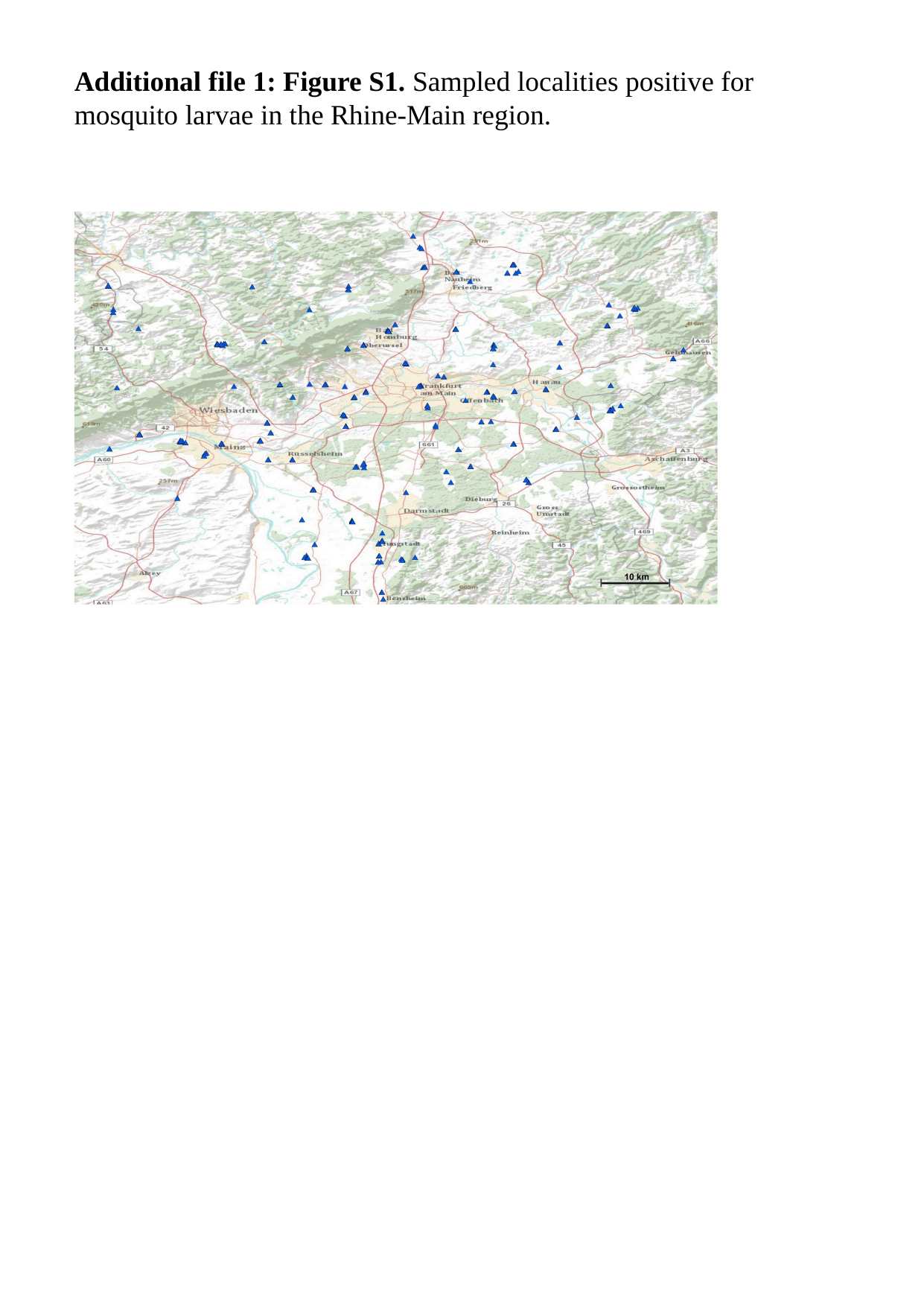

Additional file 1: Figure S1. Sampled localities positive for mosquito larvae in the Rhine-Main region.
