# Supplementary material for: Larval superiority of Culex pipiens to Aedes albopictus in a replacement series experiment: prospects for coexistence in Germany
Source: Parasit Vectors. 2018 Feb 2;11:80. doi: 10.1186/s13071-018-2665-3 (PMC5797359; doi:10.1186/s13071-018-2665-3)
Supplement: Supplementary file 3 — Pupal weight. Dry weight [DW, mg, mean ± 95% CI] of female Ae. albopictus and Cx. pipiens in pure and mixed cohorts in dependence of temperature and larval food regime. Line - linear regression. Dotted lines - 95% confidence interval. (PPTX 558 kb) [file 13071_2018_2665_MOESM3_ESM.pptx]

## Slide 1
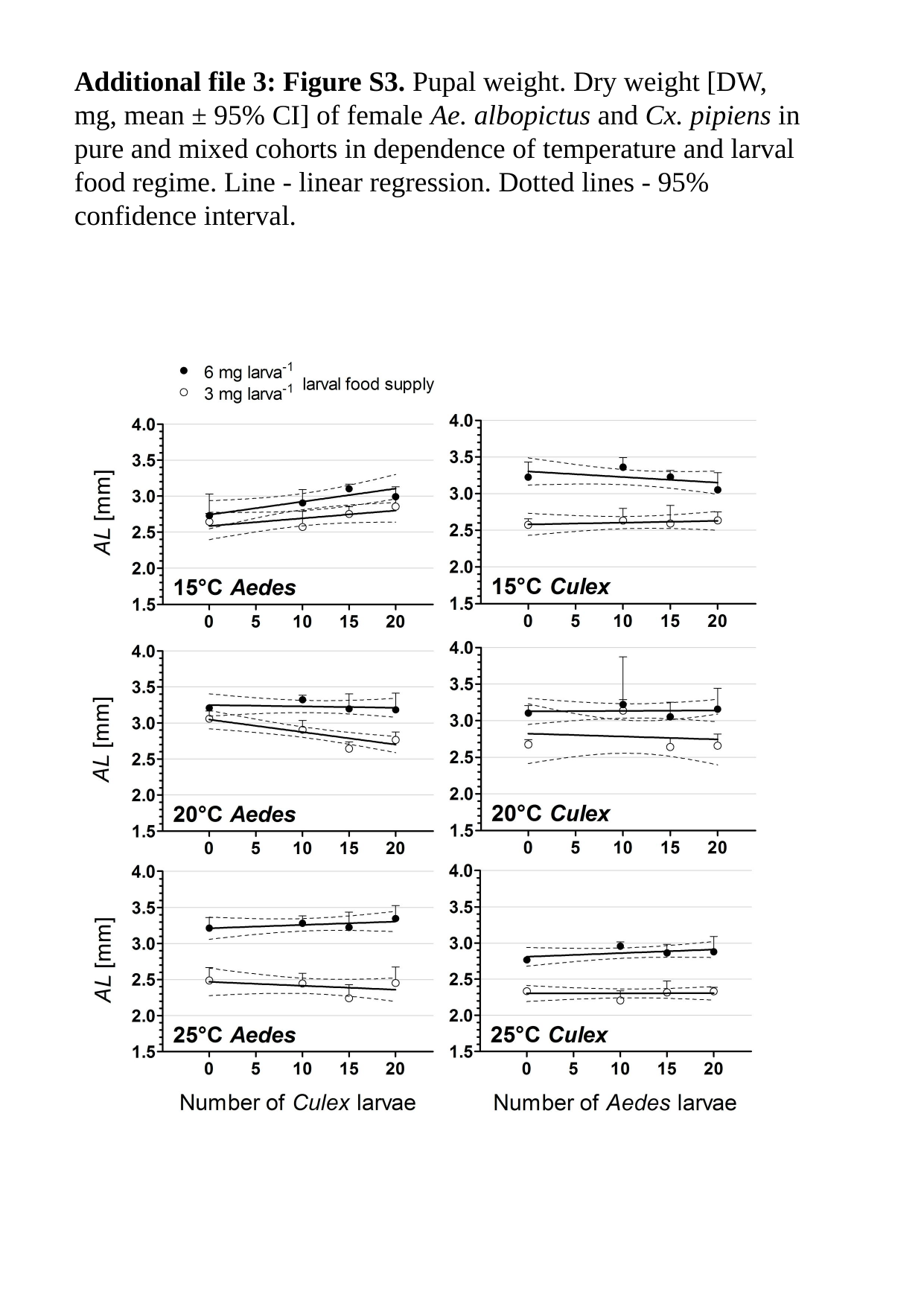

Additional file 3: Figure S3. Pupal weight. Dry weight [DW, mg, mean ± 95% CI] of female Ae. albopictus and Cx. pipiens in pure and mixed cohorts in dependence of temperature and larval food regime. Line - linear regression. Dotted lines - 95% confidence interval.
