# Supplementary material for: Larval superiority of Culex pipiens to Aedes albopictus in a replacement series experiment: prospects for coexistence in Germany
Source: Parasit Vectors. 2018 Feb 2;11:80. doi: 10.1186/s13071-018-2665-3 (PMC5797359; doi:10.1186/s13071-018-2665-3)
Supplement: Supplementary file 4 — Parameter-specific relative crowding coefficients. Relative crowding coefficient values for female and male Ae. albopictus (RCCAe) and Cx. pipiens (RCCCx) based on biomass accumulation rate (BA), pupal dry weight (DW), pupal abdominal length (AL) and mean pupation time (PT50) are listed for the different experimental treatments. (DOCX 15 kb) [file 13071_2018_2665_MOESM4_ESM.docx]

**Additional file 4: Table S1. Parameter-specific relative crowding coefficients**. Relative crowding coefficient values for female and male *Aedes albopictus* (RCC_Ae_) and *Culex pipiens* (RCC_Cx_) based on biomass accumulation rate (BA), pupal dry weight (DW), pupal abdominal length (AL) and mean pupation time (PT_50_) are listed for the different experimental treatments.

| **Larval exposure** | | **BA-RCC_Ae_** | **BA-RCC_Cx_** | **DW-RCC_Ae_** | **DW-RCC_Cx_** | **AL-RCC_Ae_** | **AL-RCC_Cx_** | **PT_50_-RCC_Ae_** | **PT_50_-RCC_Cx_** |
| --- | --- | --- | --- | --- | --- | --- | --- | --- | --- |
| ***female*** |  |  |  |  |  |  |  |  |  |
| 6 mg larva^-1^ | 15 °C | 1.33 | 1.62 | 1.53 | 1.37 | 1.65 | 1.26 | 1.52 | 1.38 |
|  | 20 °C | 1.21 | 1.72 | 1.69 | 1.23 | 1.42 | 1.46 | 1.46 | 1.43 |
|  | 25°C | 0.92 | 2.27 | 1.52 | 1.38 | 1.43 | 1.46 | 2.08 | 1.01 |
|  |  |  |  |  |  |  |  |  |  |
| 3 mg larva^-1^ | 15 °C | 1.87 | 1.12 | 1.27 | 1.65 | 1.61 | 1.30 | 1.31 | 1.59 |
|  | 20 °C | 1.47 | 1.43 | 1.50 | 1.39 | 1.46 | 1.43 | 1.38 | 1.53 |
|  | 25 °C | 1.95 | 1.07 | 1.35 | 1.55 | 1.36 | 1.54 | 1.13 | 1.84 |
| ***male*** |  |  |  |  |  |  |  |  |  |
| 6 mg larva^-1^ | 15 °C | 1.09 | 1.93 | 1.71 | 1.22 | 1.44 | 1.45 | 1.61 | 1.30 |
|  | 20 °C | 1.16 | 1.82 | 1.62 | 1.29 | 1.31 | 1.60 | 1.64 | 1.28 |
|  | 25 °C | 0.91 | 2.31 | 1.50 | 1.39 | 1.41 | 1.48 | 2.25 | 0.93 |
|  |  |  |  |  |  |  |  |  |  |
| 3 mg larva^-1^ | 15 °C | 1.72 | 1.22 | 1.30 | 1.60 | 1.50 | 1.39 | 1.34 | 1.56 |
|  | 20 °C | 1.46 | 1.44 | 1.82 | 1.14 | 1.29 | 1.61 | 1.15 | 1.82 |
|  | 25 °C | 1.54 | 1.36 | 1.41 | 1.47 | 1.40 | 1.49 | 1.38 | 1.51 |
